# Hydrogen Dynamics in Cyanobacteria Dominated Microbial Mats Measured by Novel Combined H_2_/H_2_S and H_2_/O_2_ Microsensors

**DOI:** 10.3389/fmicb.2017.02022

**Published:** 2017-10-18

**Authors:** Karen Maegaard, Lars P. Nielsen, Niels P. Revsbech

**Affiliations:** Section for Microbiology, Department of Bioscience, Aarhus University, Aarhus, Denmark

**Keywords:** sulfate reducing bacteria, methanogens, survival, fermentation, sediment, microsensor

## Abstract

Hydrogen may accumulate to micromolar concentrations in cyanobacterial mat communities from various environments, but the governing factors for this accumulation are poorly described. We used newly developed sensors allowing for simultaneous measurement of H_2_S and H_2_ or O_2_ and H_2_ within the same point to elucidate the interactions between oxygen, sulfate reducing bacteria, and H_2_ producing microbes. After onset of darkness and subsequent change from oxic to anoxic conditions within the uppermost ∼1 mm of the mat, H_2_ accumulated to concentrations of up to 40 μmol L^-1^ in the formerly oxic layer, but with high variability among sites and sampling dates. The immediate onset of H_2_ production after darkening points to fermentation as the main H_2_ producing process in this mat. The measured profiles indicate that a gradual disappearance of the H_2_ peak was mainly due to the activity of sulfate reducing bacteria that invaded the formerly oxic surface layer from below, or persisted in an inactive state in the oxic mat during illumination. The absence of significant H_2_ consumption in the formerly oxic mat during the first ∼30 min after onset of anoxic conditions indicated absence of active sulfate reducers in this layer during the oxic period. Addition of the methanogenesis inhibitor BES led to increase in H_2_, indicating that methanogens contributed to the consumption of H_2_. Both H_2_ formation and consumption seemed unaffected by the presence/absence of H_2_S.

## Introduction

Photosynthetic microbial mats are highly diverse communities both physiologically and phylogenetically. The mats are stratified communities where different functional groups exploit the microniches created by steep gradients in light and chemistry. They form on top of solid substrates where the photosynthetic primary producers fuel heterotrophic and chemolithoautotrophic bacteria. Environments with little or no disruption from benthic fauna are a prerequisite for formation of microbial mats, and they are thus best developed where fauna is absent such as in hypersaline and high-temperature environments (Stal, 1995; Fenchel, 1998; Bolhuis et al., 2014).

Photosynthetic microbial mats are autotrophic and thus reduce CO_2_ to organic matter while oxidizing an electron donor. Under certain conditions they may, however, also produce H_2_. Hydrogen production by photosynthetic bacteria has been reported numerous times (Gest et al., 1962; Benemann and Weare, 1974; Warthmann et al., 1992; Moezelaar and Stal, 1997; Hoehler et al., 2001; Burow et al., 2012; Lee et al., 2014; Nielsen et al., 2015), and such microbial H_2_ production has recently gained considerable interest due to its potential use in renewable energy supply (Lee et al., 2010).

Photosynthetic bacteria can produce H_2_ by three different pathways: as a by-product during nitrogen fixation (Benemann and Weare, 1974; Warthmann et al., 1992), by hydrogenases during anaerobic fermentation (Moezelaar and Stal, 1997; Burow et al., 2012), and by photoproduction through the action of hydrogenases when there is an excess of energy (Warthmann et al., 1992; Appel et al., 2000). Hydrogen is a favorable electron donor for aerobic and anaerobic respiration as well as for anoxygenic photosynthesis (Drutschmann and Klemme, 1985; Hoehler et al., 1998). Even in anoxic environments the H_2_ concentration is normally kept at few nanomolar levels due to the tight coupling between production and consumption (Lovley et al., 1982; Hoehler et al., 1998; Stams and Plugge, 2009). A substantial H_2_ accumulation has, however, been observed in the cyanobacterial layer of both hypersaline (Burow et al., 2012), marine (Nielsen et al., 2015) and hot spring (Anderson et al., 1987; Revsbech et al., 2016) microbial mats during dark incubations following periods with illumination. The H_2_ production in photosynthetic mats has been suspected to be due to nitrogenase activity, but the H_2_ production in hypersaline mats seems to be mainly due to fermentation, where the photosynthetic bacteria ferment the photosynthate fixed during the day as a nighttime metabolism (Hoehler et al., 2001; Burow et al., 2012; Lee et al., 2014). The simultaneous production of other fermentation products supports this hypothesis (Burow et al., 2012; Lee et al., 2014). Analysis of the gene expression of bidirectional hydrogenases pointed to cyanobacteria of the genus *Microcoleus* as the main hydrogenogens in these mats (Burow et al., 2012; Lee et al., 2014). Sulfate is a dominant terminal electron acceptor in marine sediment (Jørgensen, 1982) and the sulfate reducing bacteria *Desulfobacterales* have been identified as the dominant hydrogenotrophs in hypersaline microbial mats (Burow et al., 2014). In hypersaline and marine sediments sulfate reduction typically outcompetes methanogenesis due to high sulfate concentrations. Some methane production has been observed in saline photosynthetic mats (Hoehler et al., 2001), but methanogenesis appeared to be an unimportant H_2_ sink as compared to sulfate reduction in a thoroughly investigated hypersaline mat (Burow et al., 2012, 2014). It is generally assumed that the upper limit for sulfate reduction and hence sulfide production is governed by the depth of O_2_ penetration, although some reports suggest that sulfate reduction may occur under oxic conditions (Canfield and Des Marais, 1991; Fründ and Cohen, 1992). During the day when oxygenic photosynthesis is producing O_2_, sulfate reduction will thus be confined to the deeper anoxic part of the sediment. After darkening when O_2_ production is replaced with consumption, sulfide moves closer to the surface and may actually reach the very surface (Revsbech et al., 1983). Sulfate reducing bacteria may contain enzymes protecting them against reactive oxygen species, which reflect their frequent presence close to the oxic zone (Fournier et al., 2003; Dolla et al., 2006), and massive presence of especially *Desulfonema* has been observed in oxic mat layers sampled during illumination (Minz et al., 1999). Occurrence of nighttime sulfate reduction in layers that were oxic during the day may thus be due to either migration of sulfate reducing bacteria into the formerly oxic zone or resumption of activity by resident sulfate reducers. Tolerance to reactive oxygen species has also been found in methanogens (Horne and Lessner, 2013; Jasso-Chávez et al., 2015).

In this study the H_2_ dynamics in a photosynthetic microbial mat were described by applying combined H_2_ – H_2_S and H_2_ – O_2_ microsensors, yielding fine scale measurements and aligned profiles in this system. The alignment of profiles measured during light-dark cycles allowed for accurate positioning of the H_2_ producing layer relative to the photosynthetically active layer that was oxic during illumination, and also to the occurrence of free sulfide. The application of these sensors was essential, as previous data seem to show that the main H_2_ producing zone in cyanobacterial mats is at the lower boundary or even below the photosynthetic zone (e.g., Figure 7 in Nielsen et al., 2015), and we hypothesized that this was due to erroneous alignment of profiles measured with different sensors.

## Materials and Methods

### Sampling Site

Samples were collected from Løgstør Bredning in Limfjorden close to the village of Aggersund (57°00′04.4″N 9°17′18.6″E). The place is subject to changing water levels where the sediment may be either covered by water or exposed to air for hours or days. Due to these harsh conditions the mats can develop without being grazed by larger invertebrates. Sampling was done by coring with Plexiglas cylinders having inner diameters of 3.6 cm and a length of 8 cm. Sampling was done in November 2014, January 2015, June 2015, September 2016, and December 2016. During each sampling campaign ∼10 samples were collected and selected cores with well-developed cyanobacterial communities were profiled.

Samples were kept moist and stored at 5°C under a halogen lamp (100 μmol photons m^-2^ s^-1^) with a timer mimicking a diurnal cycle until experiments could be conducted in an aquarium kept at 20°C. Experiments were conducted between 1 and 17 days after sampling.

### Sensors

A combined H_2_ and H_2_S microsensor was applied for measuring H_2_ and H_2_S profiles in the mat. This type of sensor has been developed very recently (Maegaard et al., 2017, submitted) and allowed for perfectly aligned profiles of H_2_ and H_2_S, as both species enter the sensor through the same membrane-covered opening at the 20–50 μm wide tip. The physical design is identical to the STOX oxygen sensor (Revsbech et al., 2009), with a Clark-type H_2_ microsensor (obtained from Unisense A/S) housed in an outer sensor capillary with a tip silicone membrane. A gold anode placed in front of the H_2_ microsensor and bathed in a ferricyanide containing electrolyte (Jeroschewski et al., 1996) serves to quantify the H_2_S entering the sensor assembly and also prevents H_2_S interference on the H_2_ signal. The H_2_ part of the sensors applied had a sensitivity of 1.2–7.7 pA μmol^-1^ L^-1^ and the H_2_S part of the sensors had a sensitivity of 16.3–32.5 pA μmol^-1^ L^-1^. The calibration of the sulfide part of the sensors was done in 0.1 M HCl where O_2_ had been removed by flushing with N_2_. A 20 mmol L^-1^ Na_2_S solution was prepared from a washed crystal of Na_2_Sx9H_2_O from which fresh calibration standards of 0, 50, 100, and 200 μmol L^-1^ H_2_S were made. No pH profiles were recorded in the mat, and the total pool of dissolved sulfide thus cannot be calculated. The pKa of H_2_S is about 7 (varying somewhat with salinity (Millero et al., 1988) and the total pool of dissolved sulfide was thus substantially higher than the measured H_2_S concentration. The calibration of the H_2_ part of the sensor was performed in water from the sampling site to which demineralized water saturated with H_2_ was added to final concentrations of 0, 50, 100, and 200 μmol L^-1^. The concentration of the H_2_ saturated stock solution was calculated according to Wiesenburg and Guinasso (1979) to contain 805 μmol H_2_ L^-1^. In addition to the combined H_2_ and H_2_S sensor, an O_2_ microsensor (Revsbech, 1989) with a tip diameter of 10 μm was applied. The O_2_ sensor was calibrated assuming O_2_ saturation (252 μmol L^-1^ at 20°C and 20‰ salinity) in the aerated aquarium. The second calibration point for the linear calibration curve was the zero reading obtained in deep anoxic sediment. Further, a combined H_2_ and O_2_ sensor was constructed according to the same principle as the combined H_2_ and H_2_S sensor, but where the H_2_S anode and ferricyanide electrolyte were substituted with a gold O_2_ cathode and bicarbonate electrolyte (Revsbech, 1989). The sensitivity was 5.7–21.8 pA μmol^-1^ L^-1^ for O_2_ and 0.7–1.0 pA μmol^-1^ L^-1^ for H_2_. The large current for O_2_ is associated with a large stirring sensitivity, amounting to an about 10% signal difference between stirred and stagnant water. Oxygen data obtained with such sensors should thus not be used for calculation of fluxes and rates near mat-water interfaces. The H_2_ and O_2_ calibrations were performed as described above. Solubility of O_2_ in water and diffusion coefficients for O_2_, H_2_ and H_2_S in water were obtained from the tables at http://www.unisense.com/files/PDF/Diverse/Seawater & Gases table.pdf.

### Profiling

The mat was placed in an aerated aquarium filled with seawater from the sampling site (20‰ salinity) with the temperature kept at 20°C. The water depth above the sample was ∼2 cm. The depth position of the sensor tips was controlled by a motorized micromanipulator connected to a PC with the data acquisition software Sensor Trace Pro (Unisense A/S). The program also served to collect the A/D converted signals from the sensors. The signal currents from all types of sensors were measured by custom-made picoammeters connected to an 8-channel A/D converter (Unisense A/S). The sensor signals were recorded for every 50 μm depth interval. The recording of a H_2_/H_2_S profile took about 5 min and recording of a H_2_/O_2_ profile took about 15 min. The times for recording profiles mentioned in the following are for the start of a profile.

### Experiments

The surface of the sediment cores was illuminated at an irradiance of 600 μmol photons m^-2^ s^-1^ in the 400–700 nm range using a halogen lamp equipped with a heat filter. This light intensity corresponds to about a third of maximum sunlight intensity at noon during mid-summer. For all H_2_ accumulation experiments the samples received 3 h of light before onset of darkness. Profiles of O_2_, H_2_ and H_2_S were recorded both during illumination and during darkness. O_2_ profiles were only recorded at steady-state light and dark conditions, whereas combined H_2_/H_2_S profiles and H_2_/O_2_ profiles were measured after 3 h of illumination and at about 20 min intervals throughout the dark period following illumination. For clarity of illustration only some of the measured profiles are shown on figures.

#### Inhibition of Sulfate Reduction and Methanogenesis in Intact Sediment Core

Sulfate reduction was inhibited by addition of 10 mmol L^-1^ Na_2_MoO_4_ during the illuminated period. The molybdate was allowed to diffuse into the mat for 2 h before illumination ceased. When the H_2_ profile during the dark period was approaching a steady-state in the presence of molybdate, 20 mmol L^-1^ 2-bromoethanesulfonic acid (BES) was added to inhibit methanogenesis (Scholten et al., 2000). Profiling ceased when a new steady state was approached.

#### Hydrogen Dynamics in Isolated Cyanobacterial Layer

To prevent migration of organisms from deeper anoxic layers to the photosynthetic zone of the mat during dark conditions, a thin slice of the mat was placed on top of seawater agar, and the mat was fastened with multiple glass needles. The about 1-mm thick mat slice was cut off the sediment core with a razor blade after 3 h of illumination. Profiling of this thin mat slice during illumination and darkness was performed as described above for the intact sediment core. The importance of sulfate reduction and methanogenesis in the separated mat was evaluated by adding sodium molybdate and BES as described above for the intact sediment core. In addition H_2_ profiling was performed on a mat slice where molybdate was added when the H_2_ concentration during dark incubation had decreased to about half of the maximum value.

#### Consumption of External Hydrogen during Illumination and Darkness

Hydrogen was added to the water phase by bubbling with a gas stream controlled by a gas mixer (Brooks Instruments 0260 with Thermal Mass Flow Controllers and Meter GF 40). Profiles were recorded during light and dark conditions until steady-state profiles were observed.

### Calculations and Modeling

Profiles of reaction rates of O_2_ and H_2_ were obtained by use of the diffusion-reaction program “Profile” (Berg et al., 1998). The porosity in this type of mat can be assumed to be 0.75 according to Glud et al. (1995). We estimated the effective diffusion coefficient in the mat (D_s_) asuming *D_s_= φ* D (Berg et al., 1998), where φ is porosity and D the diffusion coefficient in water. For the values of D for O_2_ and H_2_ we used the Unisense table values of 2.0 and 3.9.10^-5^ cm^-2^ s^-1^, respectively. The boundary conditions were a H_2_ or O_2_ flux of zero in the water column and below the consumption zone. Calculation of activity distributions from H_2_ and O_2_ profiles were only possible when the profiles were relatively stable, and we were thus unable to do calculations on data obtained just after a light-dark shift. The presence of iron minerals oxidizing and precipitating sulfide and the lack of pH data prevented modeling of H_2_S production profiles. It should be stressed that the recording of a H_2_/H_2_S profile required about 5 min, which further stresses the need of near-steady state of modeled profiles. Fick’s first law of diffusion (Crank, 1975) was applied for calculation of water – mat diffusional exchange of O_2_, assuming that the steepest gradient was in a loose surface layer with diffusion coefficient for O_2_ close to the one in water.

## Results

### Visual Impressions

The sampled mats were 1–2 mm thick rigid, dark-green structures formed on top of sandy sediment. Microscopic examination showed that *Microcoleus* sp. were dominating the oxygenic photosynthetic community, but other filamentous cyanobacteria and diatoms were present as well. The underlying sediment was black indicating sulfidic conditions. During illumination O_2_ bubbles were formed on top of the mat. A white layer of colorless sulfur bacteria was observed at the surface after several hours of dark incubation, indicating that the O_2_/H_2_S interface was at the very surface of the mat (Jørgensen and Revsbech, 1983).

### Oxygen Profiles during Light and Dark Conditions

**FIGURE 1:**
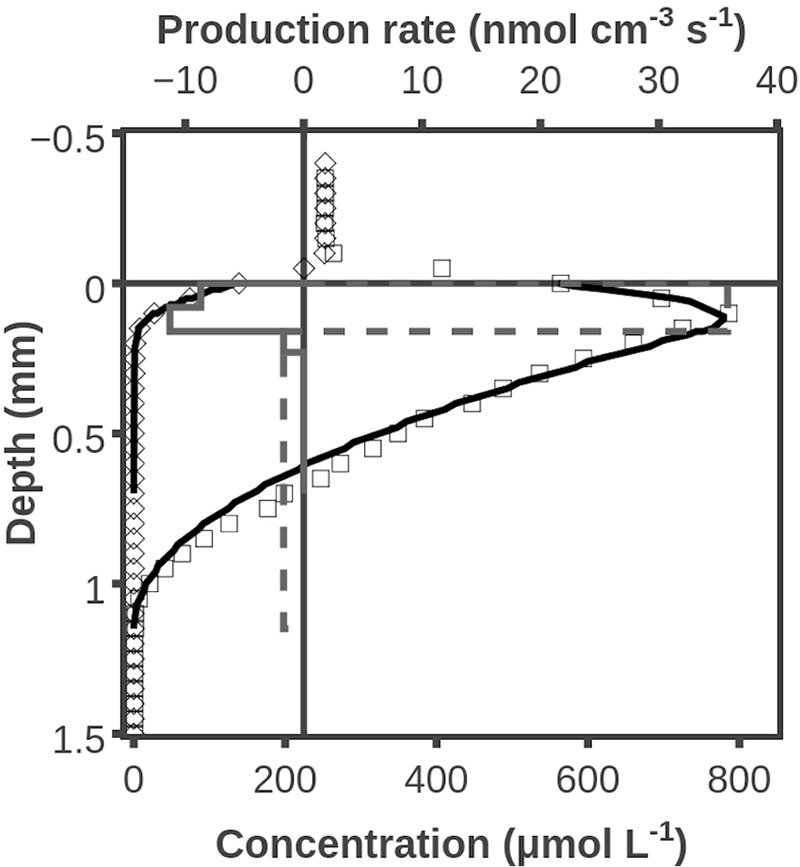
Example of O_2_ concentration profile and O_2_ production rates under dark- and light conditions. Shown are measured O_2_ concentrations during darkness (◇) and illumination (□). Also shown are modeled O_2_ concentration profiles (**―**), assuming transformation rates (during darkness: fully drawn box diagram; during illumination: with broken line) suggested by curve fitting. Data obtained by use of O_2_ microsensor.

Oxygen concentrations peaked at ∼0.1 mm during illumination and O_2_ penetrated to a depth of ∼1 mm. Modeling of the profile shown in **Figure 1** indicated a net O_2_ production zone from 0 to 0.2 mm and a net consumption zone from 0.2 to 1.1 mm. The net O_2_ production was calculated to be 0.59 nmol cm^-2^ s^-1^ and the net O_2_ consumption below 0.2 mm depth was 0.17 nmol cm^-2^ s^-1^ (**Figure 1**). Oxygen profiles were measured at 3 additional positions of the mat, and all showed O_2_ penetrations of 1.0–1.1 mm and comparable O_2_ productions in the zones with net O_2_ production. The exact figure for net O_2_ production is, however, very dependent on the diffusivity at the mat/water interface. If we assume that the steepest O_2_ gradient is in the overlying water, which will be true with a diffuse water-sediment interface, then the estimate of O_2_ production in the zone with net production (**Figure 1**) increases to 0.79 nmol cm^-2^s^-1^, and the average from all 4 profiles was 0.86 ± 0.16 (SD) nmol cm^-2^ s^-1^.

After illumination ceased a new steady state O_2_ profile was approached within 5 min, and O_2_ penetrated to a depth of only ∼0.2 mm. The modeled O_2_ consumption in the zone from 0 to 0.2 mm was 0.17 nmol cm^-2^ s^-1^ (**Figure 1**). With a similar argumentation about the steepest gradient found in a layer with a diffusivity close to the one in water, then the estimate of total diffusive uptake increases to 0.25 ± 0.04 (SD, *n* = 4) nmol cm^-2^ s^-1^. It should be stressed that the relatively large differences between surface gradient and profile modeling obtained estimates of reaction rates are due to the ill-defined boundary between mat and water. Modeling of rates in deeper layers are not subject to a similar uncertainty.

### Hydrogen and Hydrogen Sulfide Profiles under Dark Conditions

**FIGURE 2:**
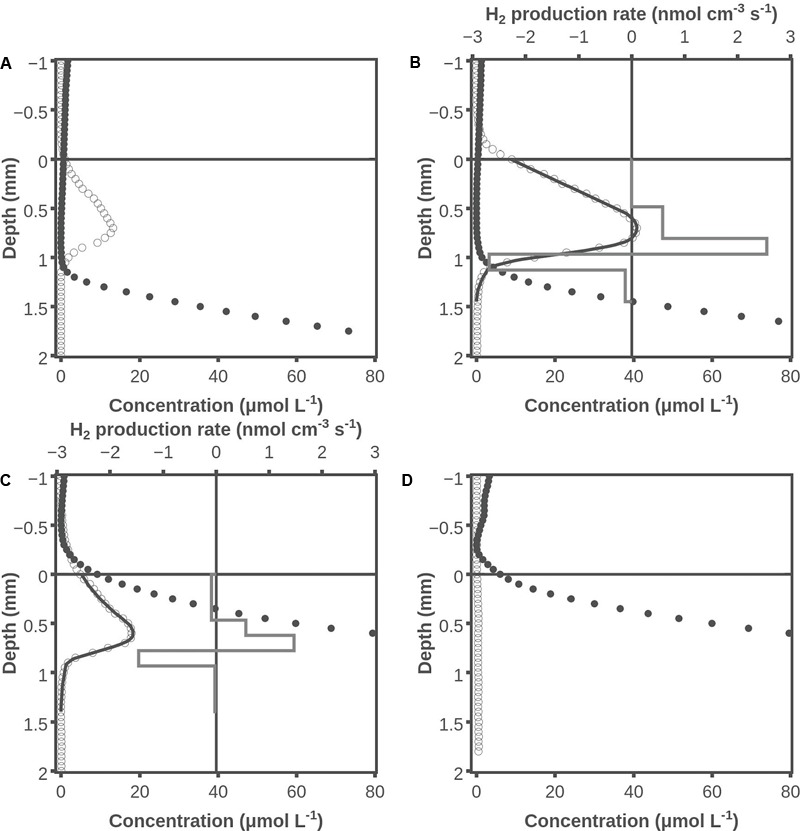
Examples of hydrogen and hydrogen sulfide concentration profiles under dark conditions. One minute after darkening **(A)**, 20 min after darkening **(B)**, 120 min after darkening **(C)**, and 240 min after darkening **(D)**. Shown are measured H_2_ concentrations (○), H_2_S concentrations (●), modeled H_2_ concentration profile (**―**), and modeled H_2_ production rate (box diagram). Data obtained by use of the combined H_2_ - H_2_S sensor.

Many sets of combined H_2_/H_2_S profiles were measured in the Aggersund microbial mat, and an example is shown in **Figure 2**. The extent of H_2_ accumulation was highly variable between samples with the maximum H_2_ levels after darkening varying from 0 to 40 μmol L^-1^ [average 12 ± 12 (SD, *n* = 13)]. We suspect that the variation was caused by differences in the species composition of the cyanobacterial community. The average depth of the H_2_ peak was 0.5 ± 0.2 (SD, *n* = 12) mm. The average H_2_ production rate was 0.031 ± 0.027 nmol cm^-2^ s^-1^ (SD, *n* = 6). Mats that appeared to be very similar could have very different levels of H_2_ accumulation, but a prerequisite for substantial H_2_ accumulation seemed to be a well-developed cyanobacterial community. The data in **Figure 2** show H_2_ accumulation higher than average, but the shapes and positions of the profiles are representative. After only 1 min of dark incubation H_2_ concentrations of up to ∼15 μmol L^-1^ were observed in the formerly oxic layer of the mat (**Figure 2A**). Hydrogen sulfide was present from a depth of ∼1.3 mm, and in this layer H_2_ was consumed as well. Hydrogen and hydrogen sulfide profiles after 20 min of darkening are shown in **Figure 2B**. Here the H_2_ accumulation peaked with a concentration of ∼40 μmol L^-1^ and H_2_S was present from a depth of ∼0.8 mm. Modeling of the H_2_ profile showed a production zone from 0.5 to 1 mm and a consumption zone from 1 to 1.3 mm. The H_2_ production in the zone with net production was 0.060 nmol cm^-2^ s^-1^ and the H_2_ consumption in the layers with measurable H_2_ and net consumption was 0.043 nmol cm^-2^ s^-1^, with the difference of 0.017 nmol cm^-2^ s^-1^ diffusing out of the mat. The peak H_2_ concentrations had decreased to ∼20 μmol L^-1^ after 120 min of darkening, and H_2_S was present at the surface of the mat (**Figure 2C**). Modeling of the H_2_ profile now showed a production zone from 0.5 to 0.8 mm and a consumption zone from 0.8 to 0.9 mm, where the net H_2_ production was 0.032 nmol cm^-2^ s^-1^ and the H_2_ consumption was 0.023 nmol cm^-2^ s^-1^, respectively. At 120 min, 0.09 nmol H_2_ cm^-2^ s^-1^ diffused out of the mat. After 240 min (**Figure 2D**), there was no H_2_ accumulation and H_2_S was present to the very surface of the mat.

### Overlap of H_2_ and O_2_ under Illumination and Darkness

**FIGURE 3:**
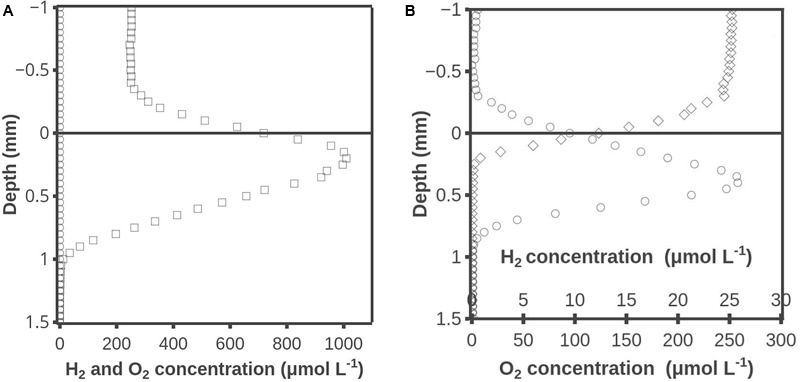
O_2_ and H_2_ concentration profiles during illumination **(A)** and 1 min after darkening **(B)**. Shown are measured H_2_ concentrations (○) and O_2_ concentrations during illumination (□) and darkness (◇). Note different scale on horizontal axis. Results obtained with the combined H_2_-O_2_ sensor.

To investigate how O_2_ affected H_2_ metabolism, the mats were analyzed with combined H_2_-O_2_ microsensors. An example of perfectly aligned data for O_2_ and H_2_ obtained with such sensors is shown in **Figure 3**. During illumination, O_2_ was produced in a zone from ∼0-0.4 mm and consumed in a zone from ∼0.4-1.0 mm, and no H_2_ was detected (**Figure 3**). The set of profiles initiated 1 min after darkening showed that O_2_ now only penetrated to ∼0.25 mm, and H_2_ was already accumulating with maximum concentrations of 26 μmol L^-1^ (**Figure 3**). It is not possible to model the rates of H_2_ metabolism as the data were obtained 1–5 min after darkening and thus are far from a steady state. However, as seen in **Figure 2** the peak of H_2_ occurs in the layer that was oxic during illumination, and the linear H_2_ profile through the ∼0.25 mm thick oxic zone indicates no net transformation in this layer. Due to sulfide poisoning of the sensor it was not possible to get H_2_ profiles for extended periods of dark incubation.

### Consumption of External Hydrogen during Light and Dark

**FIGURE 4:**
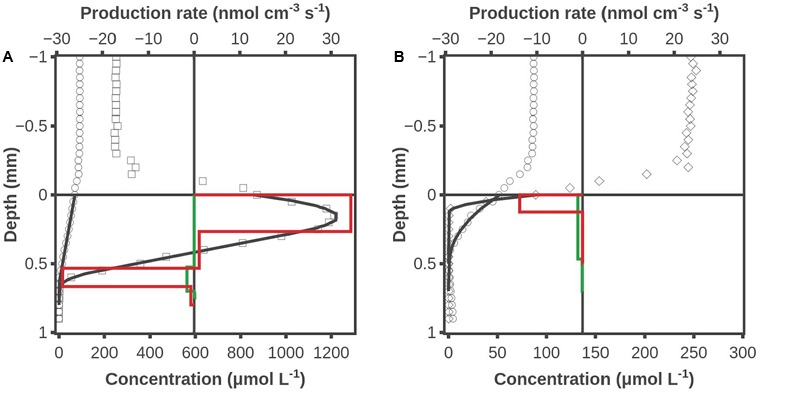
O_2_ and H_2_ concentration profiles when H_2_ was added to the water phase. **(A)** After 2 h of illumination, **(B)** after 2 h of dark incubation. Shown are measured H_2_ concentrations (○), O_2_ concentrations during illumination (□), O_2_ concentration during darkness (◇), modeled H_2_ and O_2_ concentrations (**―**), modeled H_2_ production rate (green box diagram) and modeled O_2_ production rate (red box diagram). Data obtained by combined H_2_ – O_2_ sensor.

A series of experiments were conducted where H_2_ was added to the overlying water, and an example of data from these experiments is shown in **Figure 4**. Illumination resulted in O_2_ being produced in a zone from 0 to 0.55 mm at a rate of 0.94 nmol cm^-2^ s^-1^ and O_2_ being consumed in a zone from 0.55 to 0.65 mm at a rate of 0.38 nmol cm^-2^ s^-1^. The main H_2_ consumption zone was from 0.5 to 0.7 mm, and H_2_ was consumed at a rate of 0.03 nmol cm^-2^ s^-1^ (**Figure 4**). There was no indication of any H_2_ consumption in the oxic photosynthetic zone, but a high rate of H_2_ consumption at the oxic-anoxic interface. This consumption could be due to both photosynthetic (Drutschmann and Klemme, 1985) and non-photosynthetic prokaryotes. Oxygen was consumed in a zone from 0 to 0.15 mm at a rate of 0.17 nmol cm^-2^ s^-1^ during darkness. Hydrogen was consumed down to 0.5 mm at a uniform low rate integrating up to 0.05 nmol cm^-2^ s^-1^ (**Figure 4**). It is not possible to judge if the oxic zone actually had lower activity than the anoxic zone, but modeling of the profile with zero activity in the oxic zone gave just as good a curve fit (data not shown) as the activity distribution shown in **Figure 4B**.

### Hydrogen Accumulation When Sulfate Reduction Was Inhibited by Molybdate and Methanogenesis Was Inhibited by BES

**FIGURE 5:**
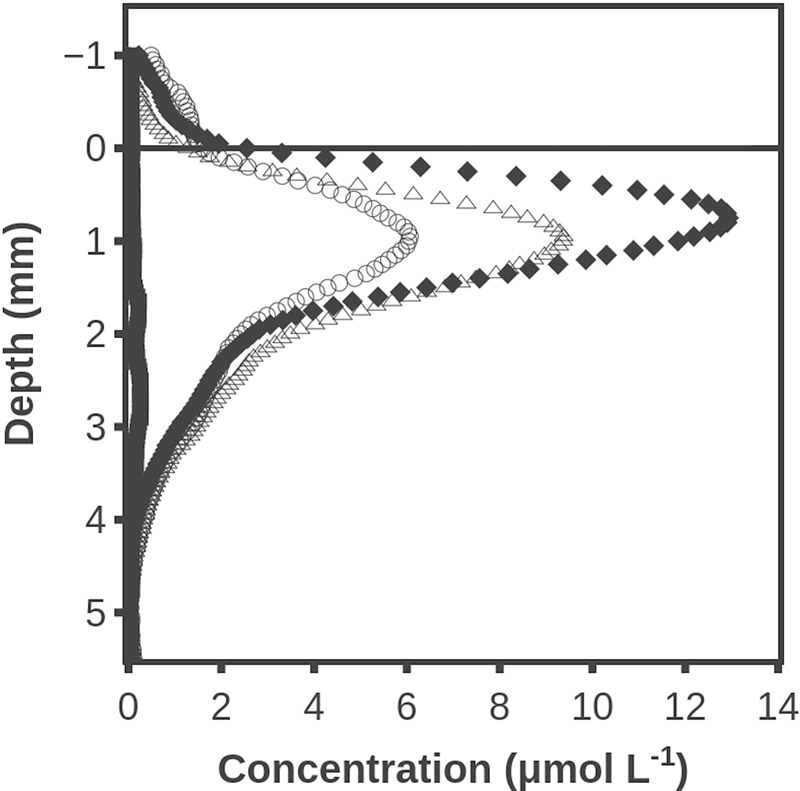
Hydrogen accumulation after darkening when sulfate reduction was inhibited with molybdate and after 210 min where methanogenesis was additionally inhibited by addition of BES. Time after darkening: 1 min (■), 120 min (◆), 210 min (○) and 240 min (△). Data obtained by a combined H_2_ – H_2_S sensor.

Substantial H_2_ concentrations persisted for a long time in the mat when sulfate reduction was inhibited by molybdate (**Figure 5**). After 120 min the H_2_ accumulation thus peaked with a concentration of ∼13 μmol L^-1^. This concentration is much lower than the levels shown in **Figure 2**, but this may be due to a less developed mat. After 210 min the maximum H_2_ concentration had decreased to ∼6 μmol L^-1^, and the methanogensis inhibitor BES was added. Inhibition of methanogenesis resulted in the maximum H_2_ concentration increasing to ∼10 μmol L^-1^. Hydrogen sulfide profiles are not shown since sulfide forms a complex with molybdate (Newport and Nedwell, 1988). A positive effect of BES on H_2_ accumulation was also seen in two similar experiments with an average increase in maximum H_2_ concentrations of 53 ± 9% (SD, *n* = 3).

### Hydrogen and Hydrogen Sulfide Profiles When Microbial Migration from Anoxic Layers to the Oxic Zone Was Prevented

**FIGURE 6:**
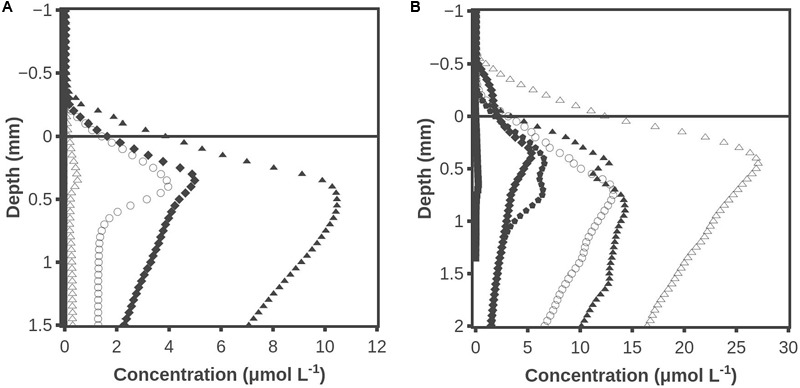
**(A)** Hydrogen accumulation when the top ∼1 mm cyanobacterial layer of the mat was placed on agar. Time after darkening: 1 min (■), 10 min (◆), 30 min (▲), 60 min (○) and 450 min (△). **(B)** Hydrogen concentration profiles when the surface ∼1 mm of the mat was placed on agar. After 165 min molybdate was added to inhibit sulfate reduction. Time after darkening: 1 min (■), 10 min (◆), 20 min (▲), 30 min (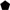), 170 min (○) and 225 min (△). Data obtained by the combined H_2_ – H_2_S sensor.

Hydrogen accumulation after darkening also occurred when the photosynthetically active part of the mat was placed on agar (**Figure 6A**). The H_2_ accumulation peaked after ∼30 min and H_2_ was barely detectable after 450 min. No H_2_S was detected at any time (data not shown). Oxygen profiles were measured at several locations and all data showed that the mat was oxic in all layers during the illumination but became anoxic below about 0.2 mm after 5 min of darkening (data not shown). In another experiment with the surface ∼1 mm of the mat placed on agar, the H_2_ accumulation peaked at ∼15 μmol L^-1^ after 20 min of darkness (**Figure 6B**). When the maximum concentration had decreased to ∼4 μmol L^-1^ at 165 min, molybdate was added to inhibit sulfate reduction. This resulted in a sizable stimulation of the H_2_ accumulation so that a maximum concentration of 27 μmol L^-1^ was reached. Sequential addition of inhibitor was also tested on a new slice of mat by adding molybdate 2 h before darkening and adding BES after 210 min of darkening. The maximum level of H_2_ of 13 μmol L^-1^ in the molybdate inhibited mat occurred after 20 min of darkening, after which H_2_ decreased to a maximum of 5 μmol L^-1^ at 195 min. When BES was added, it led to an increase of the maximum H_2_ concentration to 15 μmol L^-1^ at 315 min (data not shown).

## Discussion

When comparing stratification of chemical species and types of metabolism in compact microbial communities it is very important to align profiles obtained by different methods at the same depth scale. Various procedures have been used to get a reasonable alignment of microsensor profiles, including gluing sensors together with tips very close to each other (e.g., Revsbech et al., 1983) and visual determination of “surface” by observing the individual sensor tips during insertion. Until now only the combined O_2_/N_2_O sensor (Revsbech et al., 1988) has allowed for simultaneous measurement of two gasses in the same point. We here present the first comprehensive study of H_2_ metabolism in a microbial mat where we have totally aligned profiles of H_2_/H_2_S and H_2_/O_2_ so that interactions between H_2_, O_2_ and sulfur cycling can be precisely evaluated.

### Position of H_2_ Producing Layer

The microbial mat was oxic down to about 1 mm during illumination, but after darkening O_2_ only penetrated to about 0.2 mm. When darkened, H_2_ accumulated only within the formerly oxic zone of the intact sediment cores, where photosynthates formed during illumination were available for fermentation. The zone of net H_2_ formation as measured by the combined H_2_ – H_2_S sensor was in anoxic layers below 0.4–0.5 mm depth and thus relatively far below the depth of maximum net O_2_ production as measured by a separate O_2_ sensor. When the combined H_2_ – O_2_ sensor was used we observed, on the other hand, a perfect alignment between the concentration peak of H_2_ and photosynthetic layers below 0.2 mm that became anoxic by darkening. It thus seems that our surface definition was about 0.2 mm wrong when we estimated the surface from combined H_2_-H_2_S data. This illustrates the problems of aligning profiles from separate sensors and the strength of using sensors with dual chemical sensing as used here. By use of the two types of sensors with dual chemical sensing it was possible to determine at very high resolution how H_2_ production and consumption were associated with the presence of both O_2_ and H_2_S. The problem of aligning profiles from different sensors was in our case probably aggravated by the very different tip shapes and dimensions of O_2_ and combined sensors, where the O_2_ sensor had a slimline-tip of 10 μm and the combined sensors had blunt tips of 50 μm. Insertion of a microsensor changes the flow pattern above the mat and also the thickness of the diffusive boundary layer (Glud et al., 1994), and this effect is very dependent on sensor dimensions and shape.

### Position of H_2_ Consuming Layers

The results shown in **Figures 3, 4** were obtained by use of combined H_2_-O_2_ sensors. Microbial mat overlaid with air-saturated water was analyzed in **Figure 3**, and the dark profile initiated 1 min after darkening showed no sign of H_2_ consumption in the 0.2-mm thick oxic surface layer. Further proof for the very low H_2_ consumption potential is provided by the data in **Figure 4**, obtained after saturating the overlying water with a mixture of ∼10% H_2_ gas and air. No H_2_ consumption could be seen in the oxic 0.7-mm layer of the illuminated mat (**Figure 4A**). When the mat had been dark incubated for 2 h there was a uniform low rate of H_2_ consumption throughout the formerly oxic zone (**Figure 4B**). These data are important as it has previously been suggested that sulfate reduction may occur at high rates in oxic cyanobacterial mats (Canfield and Des Marais, 1991). The absence of any significant H_2_ oxidation potential in oxic mat counter-indicates that there are active sulfate reducers in the oxic mat, as sulfate reducing bacteria generally use H_2_ as electron donor (Jørgensen, 1982).

The data obtained with combined H_2_ – H_2_S sensors shortly after darkening, but sufficiently long after darkening to approach diffusional equilibrium, all have almost linear profiles between the H_2_ production zone and the overlying water (e.g., **Figure 2B**). This indicates that not only oxic mat layers, but also mat layers that have a recent history of being oxic are characterized by very little H_2_ consumption as illustrated by **Figure 4B**. A low H_2_ oxidation potential of oxic cyanobacterial mats was also suggested by Hoehler et al. (2002).

A high H_2_ consumption potential was found in the layer that formed the oxic-anoxic interface during illumination (**Figure 2**). This indicates that anaerobic bacteria are the main hydrogenotrophs in the mat, and this is in accordance with the findings of Lee et al. (2014) who identified sulfate reducing bacteria as the main hydrogen consumers. The zone of intense H_2_ consumption moved 0.2 mm upward from 20 to 120 min after darkening (**Figures 2B,C**), indicating that the anaerobic bacteria were able to migrate. At 120 min there was also a low rate of H_2_ consumption in the surface layers that were now highly sulfidic, indicating that some anaerobic bacteria had reached these layers by migration, or that dormant bacteria permanently inhabiting these layers had become active. The presence of sulfate reducing bacteria in oxic microbial mat has previously been reported by Minz et al. (1999). The question of migration of bacteria versus revival of inactive hydrogenotrops in the layer that was oxic during the light period was addressed by slicing off the top 1 mm and placing the slice on agar (**Figures 6A,B**). When molybdate was added to the sliced mat after 165 min of dark incubation (**Figure 6B**) there was a rapid and significant increase in H_2_ concentration, indicating that resident sulfate reducing bacteria had become active although all layers of the mat slice were oxic during the preceding light period. The effect of molybdate could also be explained by change in the fermentative pattern, but we are not aware of reports indicating such an effect of molybdate. So after >1 h of dark incubation it seems that both migration from below and revival of resident anaerobic hydrogenotrophic microorganisms caused hydrogen consumption in the surface layers of intact mats.

### Sulfate Reduction versus Methanogenesis as H_2_ Sinks

Previous investigations (Lee et al., 2014; Nielsen et al., 2015) indicate that sulfate reduction is the major hydrogenotrophic process in dark-incubated cyanobacterial mats, although methanogenesis may also occur in saline environments (Oremland and Taylor, 1978). We investigated the possible role of methanogens as hydrogenotrophs in the mat by sequentially adding molybdate and BES to both intact (**Figure 5**) and sliced mat (data not shown). Data from experiments with addition of inhibitors should always be interpreted with care as no inhibitor is totally specific. Molybdate thus complexes with H_2_S (and thereby lowers the H_2_S activity), and it also forms complexes with volatile fatty acids (VFAs) (Finke et al., 2007). BES addition caused a significant increase in H_2_ concentration, indicating that significant numbers of active methanogens were present. As expected the effect of BES was smaller than the effect of molybdate, in accordance with the conclusions of Lee et al. (2014) that sulfate reducers are the main hydrogenotrophs in such mats. The high H_2_ concentrations in a dark-incubated mat might, however, make methanogenesis locally and temporally favorable (Hoehler et al., 2002). The effect of BES in the sliced mat provides evidence that methanogens like sulfate reducers could survive long periods of oxic conditions in the mat, as previously described for a hot spring microbial mat (Revsbech et al., 2016) and soils (Angel et al., 2012).

### Absence of H_2_S from Sliced Mat

It should be noticed that sulfide did not accumulate in the sliced 1-mm mat layer during the dark incubation (**Figures 6A,B**). That might seem to counter-indicate H_2_ consumption by sulfate reduction, suggested by the increase in H_2_ after addition of molybdate, but the data in **Figure 2** may offer an explanation. Here H_2_S was found in high concentrations in layers deeper than 1.1 mm after 1–6 min (the time to record a profile was about 5 min) of darkness, and the situation was basically unchanged after 20–26 min of darkness. The mean diffusion time for a H_2_S molecule over a distance of 1 mm is less than 10 min, assuming a diffusion coefficient of 1 × 10^-5^ cm^2^s^-1^ within the mat (Jørgensen, 2000). By diffusion alone the whole mat should thus have been highly sulfidic already after a few minutes, and that points to extensive sulfide consumption within the previously oxic mat. The consumption of free sulfide may be due to oxidation and precipitation by iron and manganese minerals. The buffer capacity of the iron pool should be very high if it could consume the high sulfide flux from below in the intact mat for more than 20 min. The iron pool might thus be able to keep an isolated 1-mm top layer free of dissolved sulfide for a long time. An alternative explanation for the absence of free sulfide is oxidation by cable bacteria (Pfeffer et al., 2012). The mats from Aggersund have been found to be rich in cable bacteria (L.P. Nielsen, unpublished), and these may be able to oxidize H_2_S within the upper few mm of the mat by transporting the electrons to the surface of mat. In intact mat they may not be able to oxidize all the sulfide diffusing up from below, but the much lower amount formed in a thin slice might all be oxidized.

### Rate of H_2_ Formation as Compared to Net Photosynthesis

The rates of net H_2_ production deduced from **Figure 2B** were 0.06 nmol cm^-2^ s^-1^ when they were at the highest. This is about 10% of the rate of net O_2_ production in the same mat (**Figure 1**). Even in the presence of molybdate there is some decrease in H_2_ production with time (**Figure 5**), but the illumination periods were also limited, so the figures are roughly comparable. One O_2_ corresponds to two H_2_ molecules in terms of electron transfer, so about 5% of the reducing equivalents stored in organic compounds during the light period were liberated as H_2_. Fermentation of glucose by a *Clostridium* that was studied because of a high rate of H_2_ formation was found to yield up to 2 moles of H_2_ per mole of glucose (Jo and Kim, 2016). Assuming a loss of glucose equivalents as fast as the rate of build-up during photosynthesis, the 0.6–0.9 nmol O_2_ cm^-2^ s^-1^ corresponds to a formation rate of 0.2–0.3 nmol H_2_ cm^-2^ s^-1^. The loss rate of glucose in the dark should actually be lower than the rate of glucose production by photosynthesis in the light, and we thus also see only 20–30% of the H_2_ production calculated above. The mass balance considerations do indicate a substantial loss of photosynthates by fermentation during the dark period. This is in accordance with previous estimates of extreme rates of sulfate reduction is such mats (Jørgensen and Cohen, 1977), as the sulfate reducing bacteria utilize the fermentation products.

Based on our data we cannot rule out that there was H_2_ production due to nitrogenase activity, which would invalidate the H_2_ budget outlined above. However, Burow et al. (2012) and Lee et al. (2014) found no effect on H_2_ accumulation in a similar mat when nitrogen fixation was inhibited. Both studies observed a production of other fermentation products as well as H_2_, which support fermentation and not nitrogen fixation as the H_2_ source. The immediate rise in H_2_ after darkening observed in our study also points to fermentation as the major H_2_ forming process, as *de novo* synthesis of the O_2_ sensitive nitrogenase would require some time.

## Conclusion

The analyzed microbial mat produced high concentrations of H_2_ by fermentation after onset of darkness. The layers that had the highest photosynthetic activity in the light and became anoxic in the dark had the highest rate of H_2_ production. Results from addition of inhibitors suggest that sulfate reducing bacteria were the main hydrogenotrophs of the mat, but that methanogenic archaea also contributed to the disappearance of the H_2_ peak. Both sulfate reducing bacteria and methanogens were apparently able to survive the high O_2_ concentrations within the mat during illumination and become active again within less than 2 h of anoxia. Oxic conditions in the mat during illumination resulted in low H_2_ consumption potential, and this low potential persisted at least 30 min after anoxia was reached during subsequent darkening. This indicates that the anaerobes in the mat could survive the oxic conditions during illumination, but in a dormant stage.

## Author Contributions

KM did all measurements and was leading in the writing of the manuscript. NR supervised the study, was active in the development of new sensors, and participated in the writing of the manuscript. LN co-supervised the study and participated in the writing of the manuscript.

## Conflict of Interest Statement

The authors declare that the research was conducted in the absence of any commercial or financial relationships that could be construed as a potential conflict of interest.
